# Reasons for poor adherence to antiretroviral therapy postnatally in HIV-1 infected women treated for their own health: experiences from the Mitra Plus study in Tanzania

**DOI:** 10.1186/1471-2458-13-450

**Published:** 2013-05-07

**Authors:** Matilda Ngarina, Rebecca Popenoe, Charles Kilewo, Gunnel Biberfeld, Anna Mia Ekstrom

**Affiliations:** 1 Department of Obstetrics and Gynaecology, Muhimbili National Hospital, P.O. Box 65561, Dar es Salaam, Tanzania; 2 Department of Public Health Sciences, Karolinska Institutet, Stockholm, Sweden; 3 Department of Infectious Diseases, Karolinska University Hospital, Stockholm, Sweden; 4 Department of Obstetrics and Gynaecology, Muhimbili University of Health and Allied Sciences, Dar es Salaam, Tanzania; 5 Swedish Institute for Communicable Disease Control (SMI), Karolinska Institutet, Stockholm, Sweden; 6 Department of Microbiology, Tumor and Cell Biology, Karolinska Institutet, Stockholm, Sweden

**Keywords:** Prevention of mother-to-child transmission, Postnatal, Antiretroviral drugs, Adherence, Tanzania

## Abstract

**Background:**

In a study of prevention of mother-to-child transmission of HIV (PMTCT) by triple antiretroviral therapy (ART) in Dar es Salaam, Tanzania (the Mitra Plus study), retrospective viral load testing revealed a high and increasing frequency of detectable viral load during follow-up for two years postnatally in women given continuous ART for their own health suggesting poor adherence. This study explored women’s own perceived barriers to adherence to ART post-delivery so as to identify ways to facilitate better drug adherence among women in need of ART for their own health.

**Methods:**

Semi-structured interviews were conducted with 23 of the 48 women who had detectable viral load at 24 months postnatally. Content analysis was used to analyze the data.

**Results:**

Most women in the study did not acknowledge poor adherence until confronted with the viral load figures. Then, however, they revealed multiple reasons for failing to adhere. They said that their motivation to take ART decreased once they had protected their children from becoming infected and successfully weaned them. Feeling well for some, and a feeling of hopelessness for others, also decreased motivation to continue ART. The overwhelming demands of everyday life, poverty and lack of empowerment also posed significant barriers to long-term adherence. The need to keep their HIV status a secret and not let anyone see them taking the drugs was another steep barrier.

**Conclusion:**

Reasons for postnatal failure to adhere by mothers put on ART for life during pregnancy included lack of motivation to continue ART after weaning the child, poverty and stigma. Projects that simultaneously address stigma, poverty and women’s lack of empowerment may be necessary for PMTCT and ART to reach their full potential. Our results indicate that the new WHO proposal to start all HIV-infected pregnant women on lifelong ART regardless of CD4 cell count needs to address the challenging realities of women in resource-poor contexts if it is to be successful.

## Background

Prevention of mother-to-child transmission (PMTCT) of HIV is an entry point to lifelong antiretroviral therapy (ART) among pregnant women found to be HIV-infected [1]. Most of these pregnant women are asymptomatic before diagnosis. So far in low-income settings, life-long ART has only been offered to pregnant women who have a low CD4 cell count but this scenario is changing [2]. In high-income countries, the use of prophylactic ART combined with elective caesarean section and avoidance of breastfeeding has almost eliminated mother-to-child transmission (MTCT) of HIV but this is not the case in resource-poor settings [1]. Earlier studies in low-income countries have shown that PMTCT, involving ART during the late stages of pregnancy and postnatally, combined with exclusive breastfeeding for six months, can reduce MTCT to between 1% and 5% at 6 months after delivery [3-7].

Maintaining adherence to ART over time is a challenge in many settings also in sub-Saharan Africa where the average 24 month retention rate in ART programs 2007–2009 was 70% [8]. A large body of research has identified social, cultural and economic barriers to antenatal and postnatal adherence to ART over time [9-13]. Low levels of adherence are problematic both because poor adherence to ART leads to virologic failure, increased risk of MTCT and a high risk of drug resistance that may require a change to more expensive treatment regimens [14-17].

Mitra Plus was an open-label, non randomized, prospective PMTCT study including 501 pregnant HIV-1-infected women in Dar es Salaam, Tanzania, initiated on triple antiretroviral (ARV) drugs from 34 weeks of pregnancy until 6 months post delivery (the breastfeeding period) that assessed MTCT and infant mortality rates. Women enrolled with CD4 cells < 200/μl were put on ART for life [4] and received significant adherence counseling and support in addition to transport incentives, free medical services including management of opportunistic infections, as well as formula or food supplements for their children after stopping breastfeeding if needed.

After the end of the Mitra Plus study (2009), viral load testing showed that, contrary to women’s self-reported adherence, a high proportion of the women who were to be on ART for life had detectable viral loads at 12 months and 24 months post-delivery.

We therefore decided to conduct semi-structured open-ended interviews to explore women’s own perceived barriers to adherence to ART post-delivery and after the cessation of breastfeeding so as to identify ways to facilitate better drug adherence among women in need of ART for their own health. This knowledge is important to enable successful scale-up of the most recent WHO guidelines for PMTCT [18] including the new proposal to start lifelong ART for HIV-infected pregnant women irrespective of CD4 cell count [2].

## Methods

### Design

This was a qualitative study using inductive content analysis. It was thought to be the best method which could elicit extensive discussions that could reveal women’s perceived reasons for poor drug adherence. Qualitative interview methods facilitate rapport while enabling the interviewer to maintain focus on the issue at hand [19,20].

### Setting

The Mitra Plus clinic is located within the Muhimbili National Hospital compound, Dar es Salaam, Tanzania. It is a small clinic with no sign on the door in order to reduce the risk of stigmatization of visitors. Women were provided with bus fare to and from the clinic. Treatment of any medical condition they had during the study period was also provided free of charge. Those who could not afford formula or food supplements for their children after stopping breastfeeding were supplied with food in form of flour, milk powder and nutritional supplements.

### Participants and sampling frame

We interviewed 23 HIV-infected women enrolled in the Mitra Plus PMTCT study with a CD4 cell count of < 200/μL who were put on ART for life and followed for 2 years postpartum. Women who had detectable viral load of >400 copies/mL at the end of 2 years follow-up were purposefully selected. The interviews were conducted between August 3rd and 24th, 2009 but the actual tracing and making of appointments was done 2 months prior to interviews as most of the women had completed their follow up period and had been discharged to HIV care and treatment centers.

Of the 501 pregnant women enrolled in the Mitra Plus study, 86 had CD4 cell counts below 200/μL at enrollment and out of these 56 women were available for follow-up for at least 2 years post-delivery. Forty-eight out of these 56 women (85.7%) had detectable viral load at the end of 24 months. All 48 were traced and invited for interviews (as they had been discharged from the project and had joined care and treatment centers for HIV); of these, 23 viremic women agreed to be interviewed and tape-recorded by signing an informed consent form. Ten women did not want to be tape-recorded and were excluded from the study as the authors had agreed to deal only with tape recorded interviews for uniformity and completeness, 8 were not able to be traced; and 7 gave no reason for declining participation.

### Data collection

A semi-structured interview guide composed of open-ended questions was pretested on 2 women in this study who were eligible but refused to be tape recorded. The guide was amended and was then used during the interviews. An in depth face to face interview at a place of choice selected by each woman was thought to be the best method for enabling women who undoubtedly felt stigmatized because of their HIV infection to express themselves freely and explain the challenges they faced [21]. The first author (MN) and one research assistant conducted the interviews. Both are females, fluent in Kiswahili and English and competent in qualitative interview. The interviews were conducted in Kiswahili by politely asking and probing the participants about their lives and their experiences of taking ART. We tried to discern whether or not they had been adherent, and if not, why not. The interviews were tape-recorded and lasted between 45–60 minutes.

During the interviews we also used follow-up case notes of the client from enrolment to date, drug dispensing records, viral loads and CD4 counts to set the time line and to help the client remember events that had occurred during and after the breastfeeding period. One to two interviews were conducted daily and field notes and a field diary were kept. Peer debriefing sessions were done after the interviews to check if any issues had risen from the discussions and to help plan for the next activity. This also helped detect when saturation had been reached.

### Data management and analysis

Data analysis began during data collection and continued to evolve throughout the research as guided by Kvale [19]. This was important as it helped to plan and shape the next interview (emergent design). Tape-recorded interviews were transcribed verbatim by the research assistant and the main researcher and thereafter, the Swahili transcripts were translated to English by the research assistant and checked by the first author. The first author went through the transcripts again and compared them with the audiotapes to make sure no part of the interview was missed.

Data was analyzed using content analysis. According to Graneheim and Lundman [22] content analysis focuses on selecting the unit of analysis, meaning unit, codes, categories and theme(s). The first and second author, an anthropologist, independently coded the transcribed data. Table 1 provides an example of meaning units, condensed meaning units and codes. They compared the two coding for consistency and consensus on data interpretation. The codes were reviewed and tested by RP and AME to verify the categories.

**Table 1:** Example of a meaning unit, a condensed meaning unit and codes

**Meaning unit**	**Condensed meaning unit**	**Codes**
I took drugs properly when I was pregnant and during breastfeeding. After that and as of today I have a lot of responsibilities that I have to wake up at 4 am and I easily forget the morning dose. But it happened when the child was around one year that I stopped taking drugs…..not a single pill for almost 6 months. I even stopped coming to the clinic. I felt that I was cured. I had gained significant weight and thought I was now ok. I also traveled to Bagamoyo and Tanga for business purposes. Then I had to bring the child to the clinic. They asked me why I had disappeared and was not taking drugs. I had no good reason.	Pregnancy and breastfeeding motivation to adherence.	Ensure baby was safe.
	Responsibilities make it easy to forget and even stop taking drugs.	Busy
		Forget to take drugs
	Stopped coming to the clinic as I felt cured	Feeling well

### Trustworthiness of the study

Trustworthiness is the ability of the study methods to capture the reality of those being studied. We deem the results of this study to be trustworthy for several reasons: women’s accounts were largely in agreement with each other even though the women did not know much of one another; data saturation was reached before all the interviews were carried out; although the women’s accounts go beyond results of previous studies they do not contradict any previous research and are in line with what we know about life in poverty. Furthermore the first author MN had worked as a clinician and researcher in the study since recruitment, participating in antenatal follow-up, delivery of some of the women and postnatal follow-up making her well acquainted with the women, and they with her. The interviews were also conducted in one of the women’s native language, Kiswahili. The trustworthiness of the data analysis was further ensured by the use of follow-up case notes, peer debriefing sessions and joint analysis of the data by a research team composed of multiple professions (physicians, experts in public health, anthropologist).

### Ethical considerations

The main study protocol was approved by the institutional review boards (IRBs) of Tanzania, National Institute for Medical Research and Muhimbili University college of Health and Allied Sciences. Women were interviewed only when they had understood the purpose of the study, signed a written consent form and were ready to be tape-recorded. Consent for making phone calls or actively tracing them at their residency was signed during enrolment.

## Results

### Participants

The majority of the participants (17/23) had primary school education and 20 of the 23 interviewed women ran small-scale businesses generating low incomes. Only three had salaried jobs – nurse, secretary, and police officer. The women were between 25 and 45 years old, and the majority (17/23) had more than one child. Eight women were married, seven were cohabiting, five were widows, two were divorced and one was single. Fifteen women had disclosed their HIV sero-status to at least one member of the family, but not necessarily to their partner (Table 2).

**Table 2:** Participants characteristics

**Id number**	**Age**	**Marital status**	**Parity**	**Disclosure**	**Profession**	**Education**	**Partner’s HIV status**
1	**35**	**married**	**4**	**no**	**house-wife**	**class 7**	**not known**
2	**38**	**married**	**1**	**no**	**nurse**	**College**	**not known**
3	**37**	**married**	**2**	**yes**	**tailor**	**form 4**	**positive**
4	**40**	**widow**	**3**	**yes**	**police**	**form 4**	**positive**
5	**33**	**widow**	**3**	**yes**	**sells food**	**class 7**	**positive**
6	**28**	**divorced**	**1**	**yes**	**saloon attendant**	**class 7**	**negative**
7	**38**	**cohabiting**	**2**	**yes**	**house-wife**	**class 7**	**negative**
8	**28**	**cohabiting**	**1**	**yes**	**hotel attendant**	**class 7**	**negative**
9	**38**	**single**	**1**	**no**	**sells food**	**class 7**	**not known**
10	**36**	**married**	**3**	**yes**	**sells fruits**	**class 7**	**not known**
11	**45**	**married**	**4**	**yes**	**sells food**	**class 7**	**not known**
12	**37**	**widow**	**2**	**no**	**gardener**	**class 7**	**not known**
13	**33**	**divorced**	**3**	**yes**	**sells food**	**class 7**	**not known**
14	**29**	**married**	**3**	**no**	**sells fruits**	**form 4**	**not known**
15	**36**	**widow**	**4**	**no**	**sells food**	**class 7**	**positive**
16	**34**	**married**	**1**	**yes**	**personal secretary**	**form 4**	**negative**
17	**32**	**widow**	**3**	**no**	**sell magazines**	**class 7**	**not known**
18	**32**	**cohabiting**	**3**	**no**	**sells food**	**class 7**	**not known**
19	**34**	**cohabiting**	**1**	**yes**	**house-wife**	**class 7**	**not known**
20	**36**	**cohabiting**	**2**	**yes**	**gardener**	**class 7**	**positive**
21	**33**	**married**	**4**	**yes**	**sells food**	**no formal education**	**not known**
22	**36**	**cohabiting**	**3**	**yes**	**business woman**	**class 7**	**not known**
23	**32**	**cohabiting**	**3**	**yes**	**watchman**	**class 7**	**positive**

Most of the interviewed women ultimately, although not initially, acknowledged that they had been non-adherent to ART at some point during the study period. They only conceded that they had missed taking pills well into the interviews when informed about their high viral loads.

### Main findings

The main reasons why women neglected to adhere to ART postnatally were grouped in 6 categories: (1) they lacked motivation after having succeeded in preventing their child from becoming infected; (2) they did not feel ill; (3) life felt hopeless; (4) living in poverty constrained their ability to adhere; (5) the demands of everyday life were overwhelming; (6) they had to hide their ART due to the stigma of being HIV-infected making it difficult to follow the drug regimen.

### Lack of motivation after saving the child

Eleven of the 23 women indicated that their motivation to take ART was high while they were still breastfeeding and protecting their children from infection despite side-effects and stigma related difficulties. Once they weaned their children and knew the baby was well, women’s incentives to adhere to ART declined dramatically; despite low CD4 cell counts. As one woman described her situation:

“I used to sleep all the time, as I was very weak. It was the same when I was breastfeeding. But I was afraid to stop taking them (the ARVs), as I didn’t want to transmit the virus to my baby. So when I stopped breastfeeding I was now sure that I cannot infect my child and I could now have drug holidays. I could stay for 3–4 days without taking the drugs. (34 years, cohabiting, primary education)

For some women it was the feeling of despair that led them to stop taking their drugs after breastfeeding.

“Yes, I always remembered [to take drugs] but there are days when I was in some dilemma so did not feel like taking them so I did not take them. The baby had already stopped breastfeeding. I can say that I had lost hope. At that time my husband had died, there were family wrangles. It got to a time where I would miss my drugs for even a week. Not that I forgot to take them, I just did not want to take them. I thought it was okay if I died. Yes, I did but I gave myself hope because of my child. My child gave me strength in that I felt I had to help him. After I stopped breastfeeding that is when I lost hope completely.” (Client started crying bitterly)(40 years, widow, secondary education)

### Feeling well

Seven women stated that they felt relatively well and therefore did not think that stopping treatment would be harmful.

“*After [pregnancy and breastfeeding] and as of today I have a lot of responsibilities; I have to wake up at 4 am and I easily forget the morning dose. But it happened when the child was around one year that I stopped taking drugs-- not a single pill for almost 6 months. I even stopped coming to the clinic. I felt that I was cured. I had gained significant weight and thought I was now okay. I also traveled to Bagamoyo and Tanga for business purposes. Then I had to bring the child to the clinic. They asked me why I had disappeared and was not taking drugs. I had no good reason.” (32 years, cohabiting, primary education)*

Another woman who also felt it was difficult to take the drugs when she was not feeling sick noted that it was extra difficult to adhere when her children asked “Why do you take drugs every day, what are you suffering from?” (40 years, widow, secondary education). This reluctance to take the drugs even when feeling well was, predictably, more common among the women who had not disclosed their status and who were poorer and therefore dependent on their families for survival.

### Feeling of hopelessness

As most of the women were diagnosed to be HIV infected when they were screened at the antenatal clinic they had no time to prepare themselves psychologically and adapt to a new way of life including ART adherence despite the counseling they got. Four women spoke on how they lost hope in life and were less interested in taking ART especially after saving the child as one of them said;

“There were times when I wondered of what benefit I was to be alive. My husband died and he left me pregnant and maybe I could have been happy taking care of the child but the baby also died. I remained alive just to take this medicine how can that be?”(37 years, widow, primary education,)

### Poverty and the constraints it has on adherence

Fifteen of the 23 interviewed women explained how difficult it was for them to incorporate all of the demands of PMTCT including adherence to ART for life into lives already burdened by poverty and the struggle to survive. Most of the women in this study had only primary school education and low income if any. Some, for example, had small stands from which they sold food, earning them enough income for one or two meals a day. Only three women had formal employment. The other twenty were dependent on their small businesses, husbands or partners to complement their meager irregular incomes for food, formula, shelter, clothing and other basic needs in life. As one woman said;

“I was pregnant, sick, diagnosed to be HIV infected, partner runs away from me and had no source of income. The care and service that I got from this clinic was the one that kept me alive…” (32 years, cohabiting, primary education)

Not having money for food was a common reason for women to miss taking their drugs both because the ARVs make them feel more hungry, experience more side effects and also because they had been informed to take the drugs with food. As this woman complained;

“*They tell us that we should eat fruits and vegetables and here in Dar es Salaam one has to buy all these things. They should maybe give us some assistance on this. At the moment a day may even pass without a meal and one is not even able to afford an orange.” (37 years, widow, primary education).*

The women explained how important it was for them to have enough food to eat so that they could take their drugs; otherwise the drugs made them weak and they would hesitate to take the next dose if they had an empty stomach.

“If for example one decides to take the ARVs before eating it becomes difficult to even walk. One feels dizzy but if one has eaten well then takes the drugs then you don’t feel the effects at all.” (38 years, cohabiting, primary education)

The overriding impression was that the women were disempowered vis-a-vis both their relatives and male partners. Many were entirely dependent on their partner for financial support and with minimal negotiation power. Disclosure of HIV status to their partners or relatives, that potentially could have supported the women to take ART was associated with a strong, and apparently realistic, fear of abandonment and only a minority had disclosed to their families, leaving them very much alone in their struggles to be good mothers while also taking care of their own health and daily survival struggles, being provided drugs but no food to take the pills with.

### The unceasing demands of daily life

Ten women described how in the long run and mostly after they had stopped breastfeeding they got seriously involved in their normal duties and could easily forget to take their drugs or could not find time to go to the clinic for drug refills as follows;

“There are days I forget to carry my drugs with me and I have to wait till evening to take them. No, I am not less keen [after I stopped breastfeeding] but maybe I am just too overwhelmed with work such that I may remember about the drugs at 10 am and I will be an hour late. (38 years, cohabiting, primary education)”

“I did not have time to go get the drugs when I was employed. I used to hide and go to get the drugs but when I got here they had either closed or the queue was too long and I could not wait because I had the keys for my employers’ house. Sometimes I would ask the nurse to serve me first but the other clients would complain.” (28 years, cohabiting, primary education)

A few women said that they had many little things in their lives that all together would make them too tired to take drugs.

“Sincerely speaking I cannot really pick on one thing that was disturbing me. I just had the normal hardships of life but I cannot pick on one specific thing that bothered me. Maybe taking care of my handicapped child could have taken a toll on me. I have been hospitalized four times with her, the first time she was operated for cleft lip and palate.” (33 years, divorced, primary education)

Another woman said;

“But I’m a human being. Do you think I like forgetting to take my drugs? Really sometimes a day or two would pass without taking drugs.” (36 years, cohabiting, primary education.)

Some women explained that they had to travel frequently and cited this as the reason for poor drug adherence. So most of the women were very mobile and this tended to interfere with proper drug adherence since they sometimes run out of the drugs, forget to take them on a trip, or got motion sickness while travelling, which prevented them from taking the pills as one woman says;

“There is a time when my mother was sick and I travelled to my mother’s place. I stayed there for one month and I ran out of the medicine.”(45 years, married, primary education)

### The effect of HIV related stigma on drug adherence

Ten women spoke explicitly about stigma. Because HIV infection is still highly stigmatized in Tanzania, women are reluctant to let others see any activity that could disclose their HIV status, including taking medication, breastfeeding exclusively (as people are used to mixed feeding), or going to the clinic to get drugs. As one woman described being HIV positive said,

“*This is a secret disease. You should not go around spreading news that you are infected because once people get to know that you are infected they discriminate you. People try to avoid you and would prefer to stay far from you.” (33 years, married, no formal education)*.

The stigma of HIV arises in part because people in the society associate HIV infection with promiscuity and hence considers it shameful, sinful, and even a punishment from God.

“By the time I got tested, people thought that if one was HIV positive then they were prostitutes…..then I would have felt ashamed and embarrassed [to disclose my status]”(35 years, married, primary education)

Since most of the people in Dar es Salaam are poor and tend to live in very close contact with their extended family and with neighbors, keeping a positive HIV status secret is very difficult. Most women with small children are dependent on others for support and childcare, so having to take drugs secretly at definite times and having to exclusively breastfeed sometimes posed an insurmountable challenge. Many women related how they made considerable efforts to hide that they were on ART.

“I tried my level best not to forget taking my drugs. At times the house would be full of guests but I would just find a way to escape with water from the sitting room into the bedroom where I could take my medicine in private. I always made sure that not more than an hour passed past the time I was supposed I take my next dose. Most of the time while at the village I wouldn’t take drugs at the appropriate time due to the presence of people. This would sometime force me to swallow them without water and drink water afterwards.” (33 years, married, no formal education)

Women felt forced to constantly come up with new lies in order to conceal their practices of PMTCT and adherence to ART while still accepting the support they needed from other family members as one woman said;

“*I told [my mother] that I wanted to stop breastfeeding at six months because my work involved me being around fire and that would make my breast milk too hot. If I then breastfed the baby, it would make the baby have diarrhoea. I told her that the doctor had told us that those women who do my kind of work should not breastfeed because it will cause problems for the baby. My mother understood that and agreed with me.” (33 years, divorced, primary education)*

Clearly, adherence to the ART regimen and exclusive breastfeeding posed grave problems for most of these women who had to hide their HIV status to keep their position in society, to get respect from family and neighbors, to obtaining childcare and to get support from boyfriends or husbands.

## Discussion

The majority of the HIV-infected women in this study who started ART for life during pregnancy because of low CD4 cell count had developed detectable viral load at 24 months after delivery. This finding was particularly surprising given that most of the women reported adherence and were clinically doing well. Most of the women acknowledged poor adherence and gave explanations for it after being informed about their viral load trend.

Previous studies have found that costs of transport to and from the clinic, health clinic fees, and lost wages due to long waiting times at clinics have been the main barriers to adequate ART adherence [11,13,23-25]. Our findings suggest, however, that despite provision of free ART drugs in an ideal setting with continuity among providers as well as “extras” like bus fare, free medical services, and management of opportunistic infections, it was still a challenge for the women to adhere adequately over a two-year period. We discovered that the motivation to adhere to ART dropped after cessation of breastfeeding and saving the child. The drop in motivation was compounded by poverty, the overwhelming demands of everyday life, and the fear of stigma if relatives and neighbors found out they were infected with HIV. Motivation was also affected by the fact that most women felt well and were clinically stable and had been put on ART because they were diagnosed during pregnancy. However, all the women had been informed that their CD4 cell counts were low and that they needed to adhere to ART for life. To our knowledge this is the first study to document a decrease of motivation to adhere to lifelong ART after cessation of breastfeeding.

Some of the other reasons we found for non-adherence have also been reported in previous studies: the stigma of HIV [24,26], the forced secrecy around the disease and around taking ART, general poverty, the overwhelming demands of everyday life, a general sense of hopelessness, and a sense that one is well and therefore need not follow the demanding drug regimen [11,13,15,27-30]. The gripping detail in which women described their travails, however, breathes life into the challenges to adhering to ART even among those women who need the medication for their own immediate health and survival, many of whom were single caretakers of their infants.

Most women in this study had consistently reported high ART adherence during the follow-up in the Mitra Plus study and some even kept insisting, until we showed them the viral load results. Social desirability may be particularly strong among mothers given the strong expectations they face from health care staff in terms of ART adherence and our study suggests that self-report is not a reliable measure as it may well exaggerate adherence rates. This is very important clinically as poor adherence and virologic failure have serious consequences including increased risk of morbidity and mortality and development of drug resistance which can be costly to manage in resource-limited settings [14-16].

Poverty and HIV-related stigma combined with cultural gender norms and traditions, significantly reduces women’s power to make decisions regarding their own health in this Tanzanian society, and is probably quite generalizable to many other urban sub-Saharan African settings. Women are generally less educated and often lack their own income making them dependent on men for survival [31,32]. Lack of food and crowded housing conditions are real barriers to ART adherence directly linked to poverty. Public awareness of PMTCT and the relationship to breastfeeding and early weaning makes both adherence to ART and exclusive breastfeeding a major challenge especially among the most vulnerable, least empowered women due to societal stigma associated with HIV causing neighbors and relatives to closely observe and gossip around any signs of HIV-related deviations from normal post-partum behavior. This challenge may be dealt with by introducing structural interventions aimed at alleviation of poverty, food-shortages and the hardships of women’s lives such as educational opportunities, small scale business or job-training support, more involvement of fathers and partners in pregnancy and child-care at the community level. Efforts should also be made to reduce stigma and improve compliance by protecting and promoting the client’s right to privacy and confidentiality both in the community and in healthcare settings.

### Methodological considerations

The major limitation of this study is that it was done at the end of the follow up period when women might have forgotten what really happened in the past 2 years. Case notes kept in the clinic were used to try to remind them of some events that happened as they were recorded at certain time points during their follow up period. The fact that MN worked in the project throughout the study period and was well known by the participants before the interviews could have caused some social desirability bias, where mothers answered with what they assumed would be the right thing to say, rather than what they actually practiced. However, to reduce such bias the interviewers worked to carefully ensure friendly environment to enable open discussions and only after some time told the women about their high viral loads that had raised our concern. Most women then volunteered what appeared to be truthful information regarding lack of adherence. We also underlined the separate roles of the first author of being a researcher rather than their clinician at the time of this study.

## Conclusion

Reasons for long-term ART adherence in mothers put on ART for life during pregnancy included lack of motivation to continue ART after weaning the child and protecting the child from becoming infected, stigma and poverty. Programs that simultaneously address stigma, poverty and women’s lack of empowerment, including interventions aimed at helping women to problem-solve around barriers to long-term adherence may be necessary for PMTCT and ART to reach their full potential, especially in view of the new WHO proposal to initiate ART for life in pregnant women regardless of CD4 cell count.

## Competing interests

The authors declare that they have no competing interests.

## Author’s contributions

MN conceived and designed the study, was actively involved in data collection, data management, analysis and drafted the manuscript. CK, GB and AME participated in the study design, data analysis and critically reviewed the final draft. RP was involved in data analysis and drafting the manuscript. All authors read and approved the final manuscript.

## Pre-publication history

The pre-publication history for this paper can be accessed here:

http://www.biomedcentral.com/1471-2458/13/450/prepub
